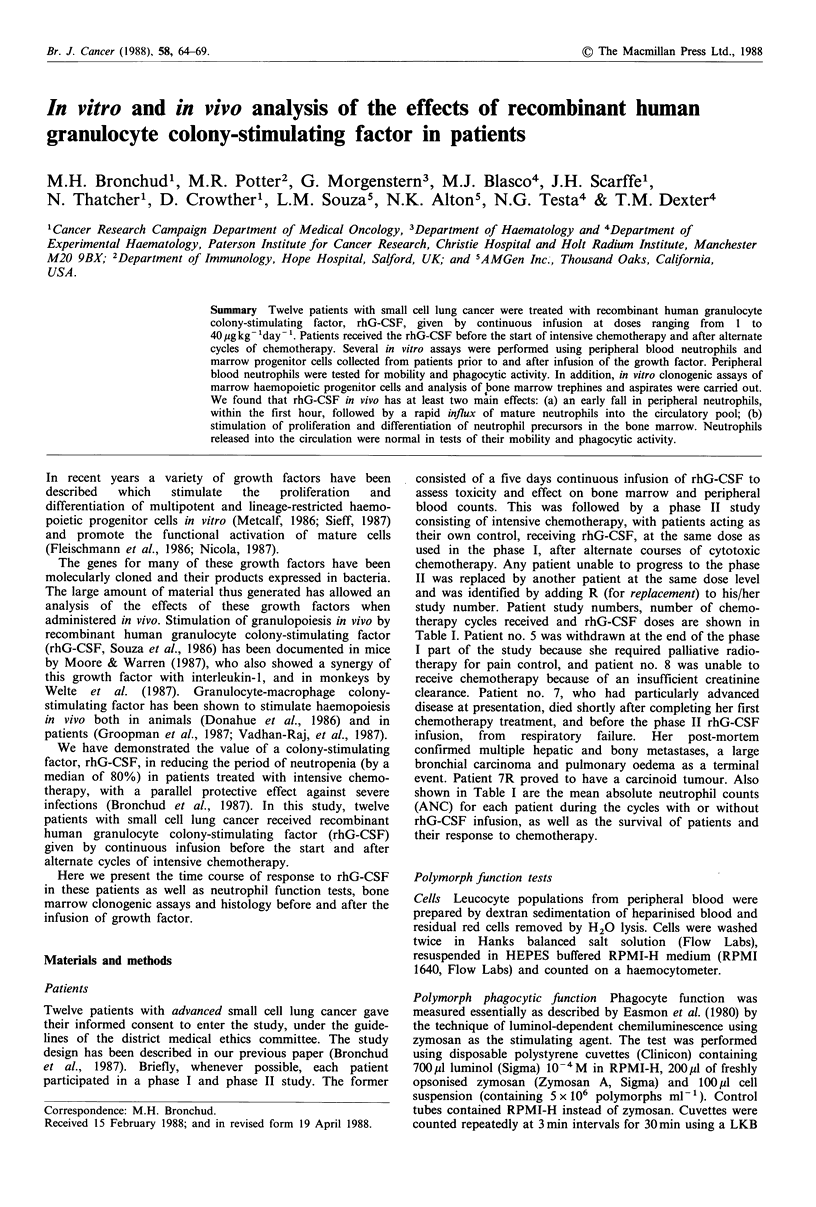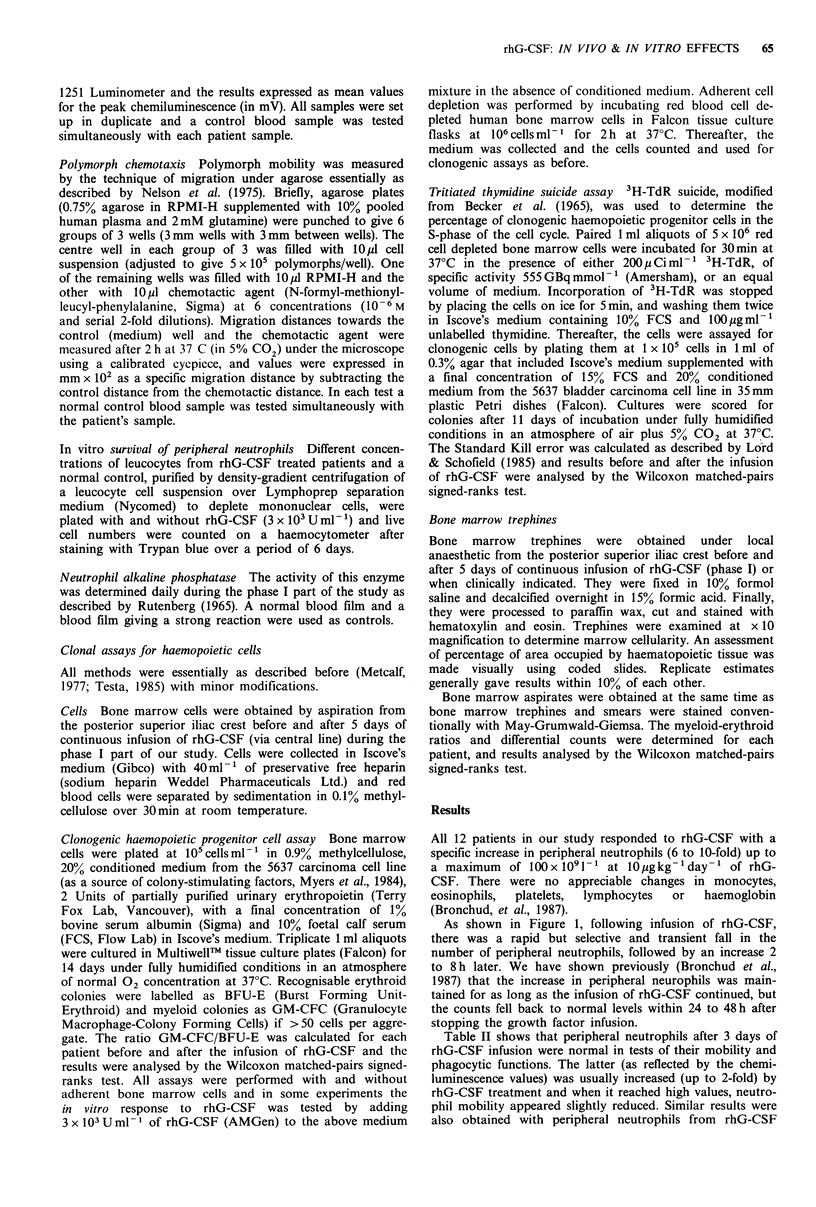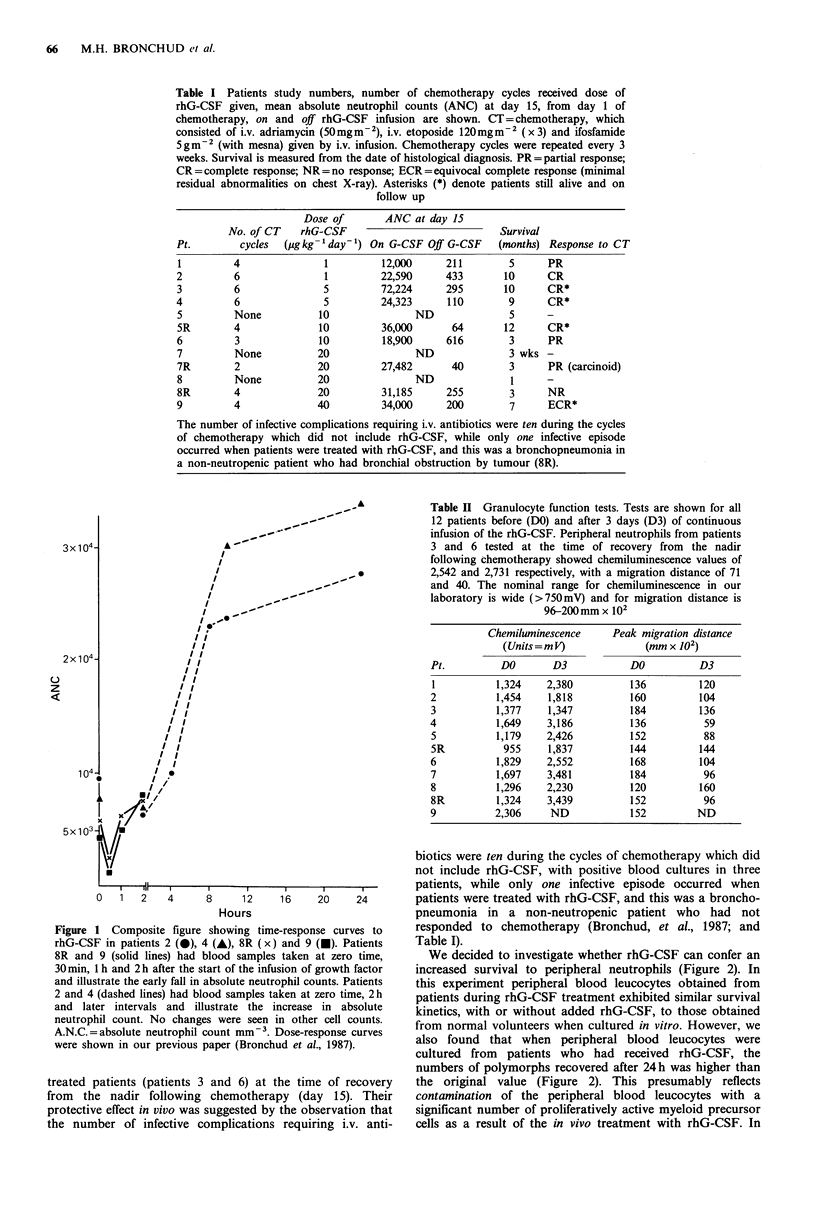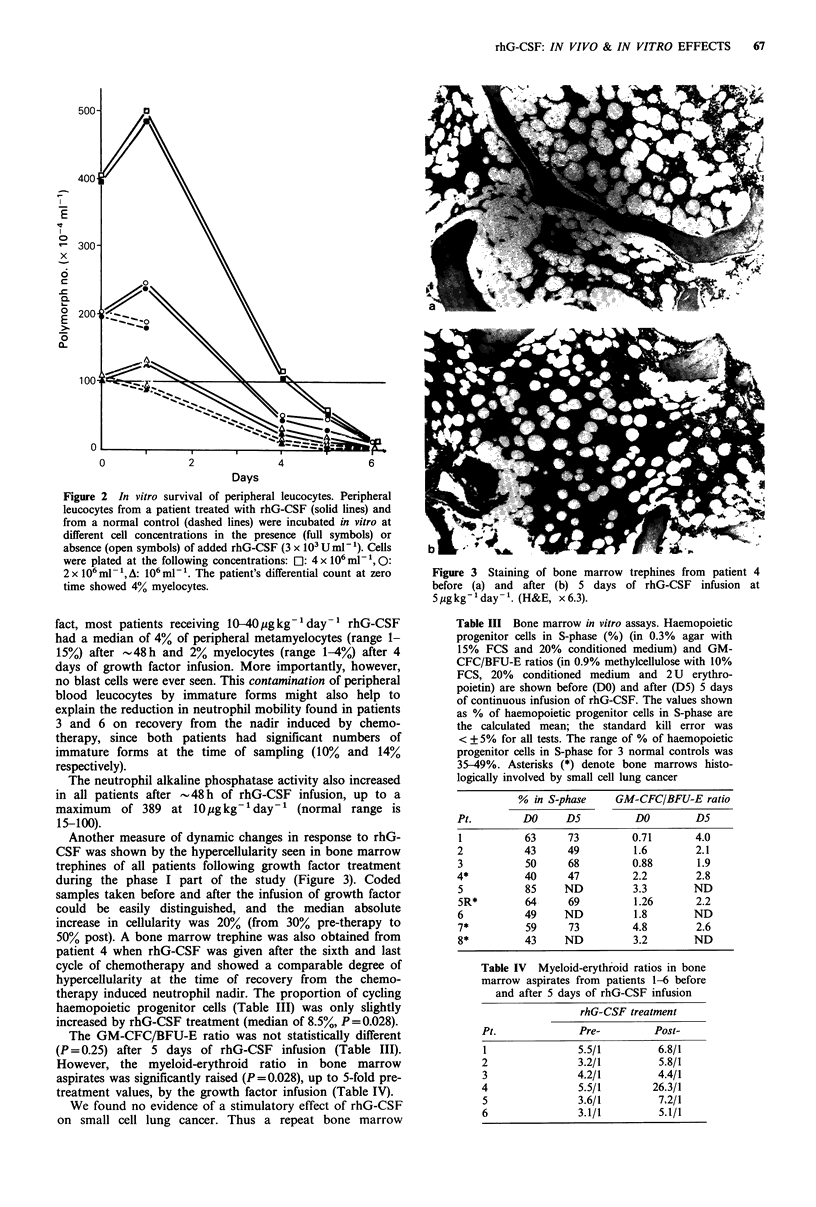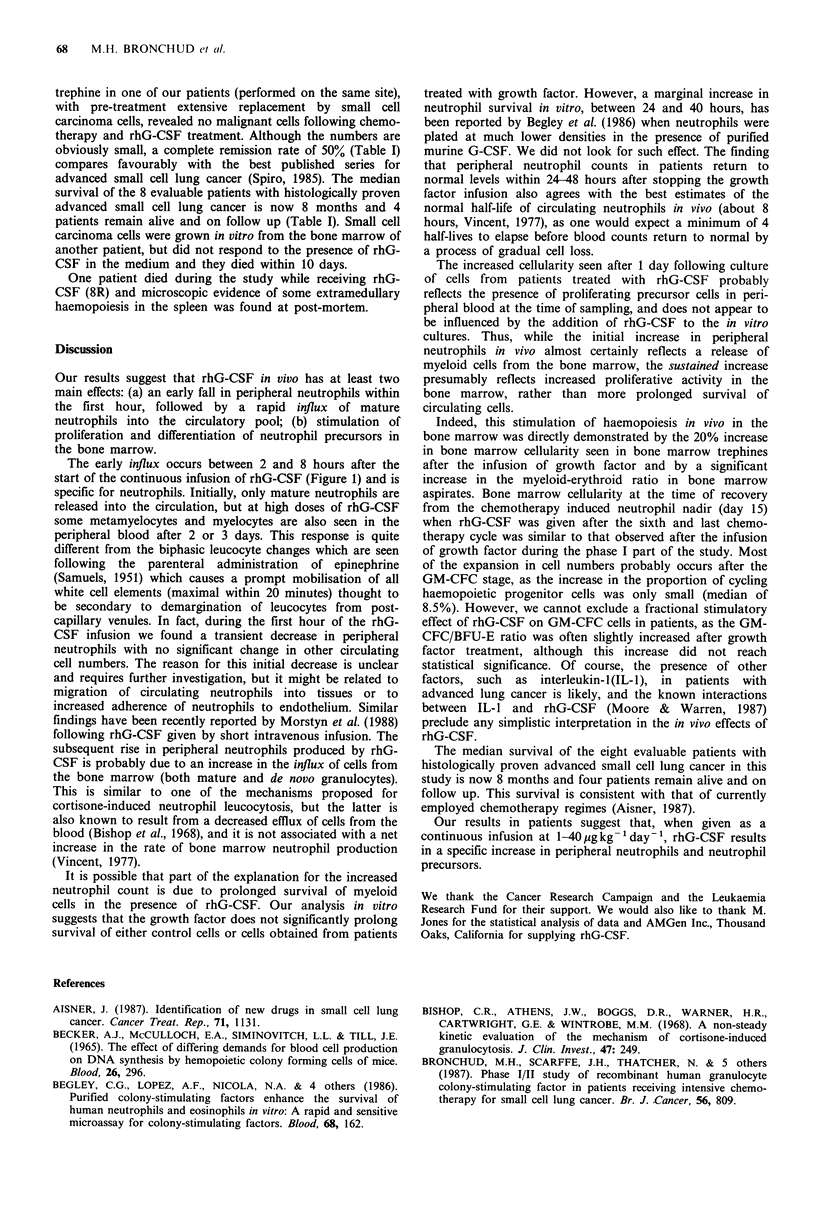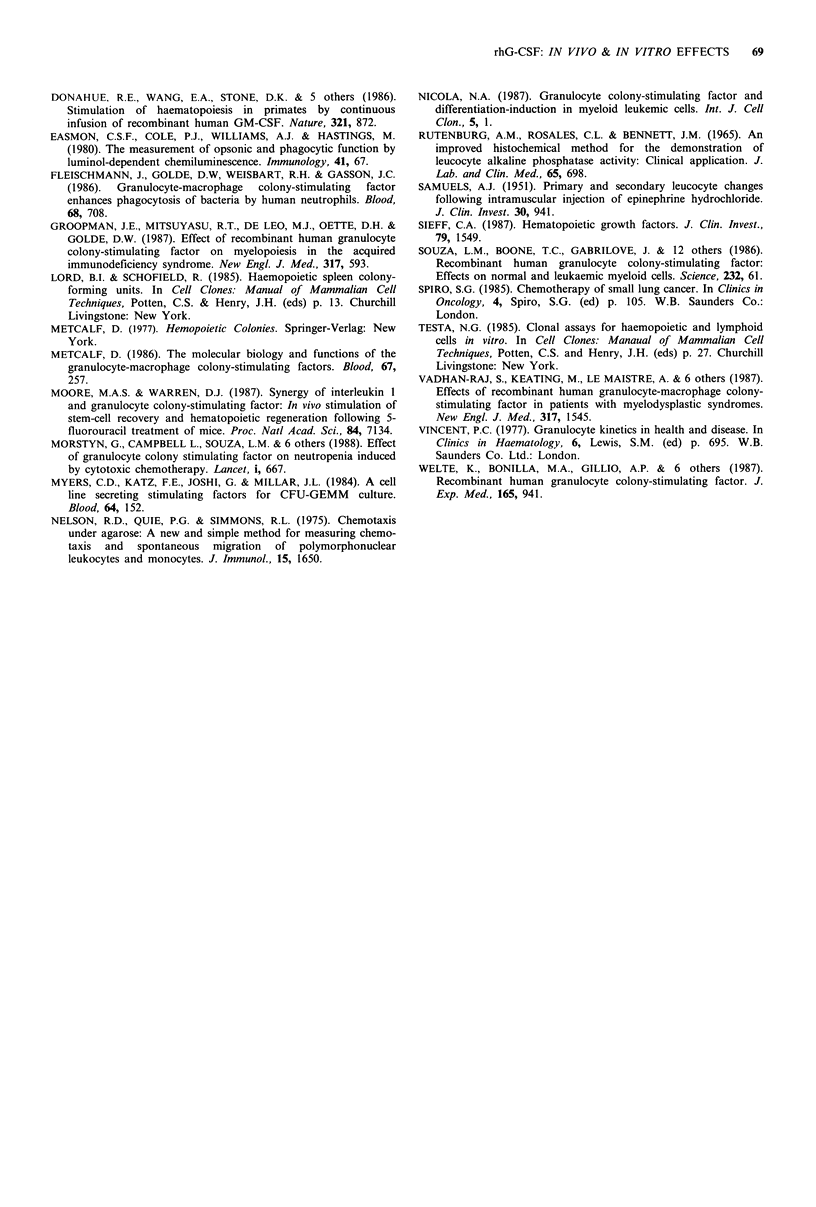# In vitro and in vivo analysis of the effects of recombinant human granulocyte colony-stimulating factor in patients.

**DOI:** 10.1038/bjc.1988.163

**Published:** 1988-07

**Authors:** M. H. Bronchud, M. R. Potter, G. Morgenstern, M. J. Blasco, J. H. Scarffe, N. Thatcher, D. Crowther, L. M. Souza, N. K. Alton, N. G. Testa

**Affiliations:** Department of Medical Oncology, Paterson Institute for Cancer Research, Christie Hospital and Holt Radium Institute, Manchester, UK.

## Abstract

**Images:**


*Br. J. Cancer* (1988), **58**, 64–69. © The Macmillan Press Ltd., 1988

### *In vitro* and *in vivo* analysis of the effects of recombinant human granulocyte colony-stimulating factor in patients

M.H. Bronchud[1], M.R. Potter[2], G. Morgenstern[3], M.J. Blasco[4], J.H. Scarffe[1], N. Thatcher[1], D. Crowther[1], L.M. Souza[5], N.K. Alton[5], N.G. Testa[4] & T.M. Dexter[4]

[1]*Cancer Research Campaign Department of Medical Oncology,* [3]*Department of Haematology and* [4]*Department of Experimental Haematology, Paterson Institute for Cancer Research, Christie Hospital and Holt Radium Institute, Manchester M20 9BX;* [2]*Department of Immunology, Hope Hospital, Salford, UK; and* [5]*AMGen Inc., Thousand Oaks, California, USA.*

**Summary** Twelve patients with small cell lung cancer were treated with recombinant human granulocyte colony-stimulating factor, rhG-CSF, given by continuous infusion at doses ranging from 1 to $40\,\mu g\,kg^{-1}day^{-1}$. Patients received the rhG-CSF before the start of intensive chemotherapy and after alternate cycles of chemotherapy. Several *in vitro* assays were performed using peripheral blood neutrophils and marrow progenitor cells collected from patients prior to and after infusion of the growth factor. Peripheral blood neutrophils were tested for mobility and phagocytic activity. In addition, *in vitro* clonogenic assays of marrow haemopoietic progenitor cells and analysis of bone marrow trephines and aspirates were carried out. We found that rhG-CSF *in vivo* has at least two main effects: (a) an early fall in peripheral neutrophils, within the first hour, followed by a rapid *influx* of mature neutrophils into the circulatory pool; (b) stimulation of proliferation and differentiation of neutrophil precursors in the bone marrow. Neutrophils released into the circulation were normal in tests of their mobility and phagocytic activity.

In recent years a variety of growth factors have been described which stimulate the proliferation and differentiation of multipotent and lineage-restricted haemopoietic progenitor cells *in vitro* (Metcalf, 1986; Sieff, 1987) and promote the functional activation of mature cells (Fleischmann *et al.*, 1986; Nicola, 1987).

The genes for many of these growth factors have been molecularly cloned and their products expressed in bacteria. The large amount of material thus generated has allowed an analysis of the effects of these growth factors when administered *in vivo*. Stimulation of granulopoiesis *in vivo* by recombinant human granulocyte colony-stimulating factor (rhG-CSF, Souza *et al.*, 1986) has been documented in mice by Moore & Warren (1987), who also showed a synergy of this growth factor with interleukin-1, and in monkeys by Welte *et al.* (1987). Granulocyte-macrophage colony-stimulating factor has been shown to stimulate haemopoiesis *in vivo* both in animals (Donahue *et al.*, 1986) and in patients (Groopman *et al.*, 1987; Vadhan-Raj, *et al.*, 1987).

We have demonstrated the value of a colony-stimulating factor, rhG-CSF, in reducing the period of neutropenia (by a median of 80%) in patients treated with intensive chemotherapy, with a parallel protective effect against severe infections (Bronchud *et al.*, 1987). In this study, twelve patients with small cell lung cancer received recombinant human granulocyte colony-stimulating factor (rhG-CSF) given by continuous infusion before the start and after alternate cycles of intensive chemotherapy.

Here we present the time course of response to rhG-CSF in these patients as well as neutrophil function tests, bone marrow clonogenic assays and histology before and after the infusion of growth factor.

### Materials and methods

### *Patients*

Twelve patients with *advanced* small cell lung cancer gave their informed consent to enter the study, under the guidelines of the district medical ethics committee. The study design has been described in our previous paper (Bronchud *et al.*, 1987). Briefly, whenever possible, each patient participated in a phase I and phase II study. The former consisted of a five days continuous infusion of rhG-CSF to assess toxicity and effect on bone marrow and peripheral blood counts. This was followed by a phase II study consisting of intensive chemotherapy, with patients acting as their own control, receiving rhG-CSF, at the same dose as used in the phase I, after alternate courses of cytotoxic chemotherapy. Any patient unable to progress to the phase II was replaced by another patient at the same dose level and was identified by adding R (for *replacement*) to his/her study number. Patient study numbers, number of chemotherapy cycles received and rhG-CSF doses are shown in Table I. Patient no. 5 was withdrawn at the end of the phase I part of the study because she required palliative radiotherapy for pain control, and patient no. 8 was unable to receive chemotherapy because of an insufficient creatinine clearance. Patient no. 7, who had particularly advanced disease at presentation, died shortly after completing her first chemotherapy treatment, and before the phase II rhG-CSF infusion, from respiratory failure. Her post-mortem confirmed multiple hepatic and bony metastases, a large bronchial carcinoma and pulmonary oedema as a terminal event. Patient 7R proved to have a carcinoid tumour. Also shown in Table I are the mean absolute neutrophil counts (ANC) for each patient during the cycles with or without rhG-CSF infusion, as well as the survival of patients and their response to chemotherapy.

### *Polymorph function tests*

*Cells* Leucocyte populations from peripheral blood were prepared by dextran sedimentation of heparinised blood and residual red cells removed by $H_2O$ lysis. Cells were washed twice in Hanks balanced salt solution (Flow Labs), resuspended in HEPES buffered RPMI-H medium (RPMI 1640, Flow Labs) and counted on a haemocytometer.

*Polymorph phagocytic function* Phagocyte function was measured essentially as described by Easmon *et al.* (1980) by the technique of luminol-dependent chemiluminescence using zymosan as the stimulating agent. The test was performed using disposable polystyrene cuvettes (Clinicon) containing $700\,\mu l$ luminol (Sigma) $10^{-4}$ M in RPMI-H, $200\,\mu l$ of freshly opsonised zymosan (Zymosan A, Sigma) and $100\,\mu l$ cell suspension (containing $5\times10^6$ polymorphs $ml^{-1}$). Control tubes contained RPMI-H instead of zymosan. Cuvettes were counted repeatedly at 3 min intervals for 30 min using a LKB

Correspondence: M.H. Bronchud.
Received 15 February 1988; and in revised form 19 April 1988.

1251 Luminometer and the results expressed as mean values for the peak chemiluminescence (in mV). All samples were set up in duplicate and a control blood sample was tested simultaneously with each patient sample.

*Polymorph chemotaxis* Polymorph mobility was measured by the technique of migration under agarose essentially as described by Nelson *et al.* (1975). Briefly, agarose plates (0.75% agarose in RPMI-H supplemented with 10% pooled human plasma and 2 mM glutamine) were punched to give 6 groups of 3 wells (3 mm wells with 3 mm between wells). The centre well in each group of 3 was filled with 10 $\mu$l cell suspension (adjusted to give $5 \times 10^5$ polymorphs/well). One of the remaining wells was filled with 10 $\mu$l RPMI-H and the other with 10 $\mu$l chemotactic agent (N-formyl-methionyl-leucyl-phenylalanine, Sigma) at 6 concentrations ($10^{-6}$ M and serial 2-fold dilutions). Migration distances towards the control (medium) well and the chemotactic agent were measured after 2 h at 37 C (in 5% $CO_2$) under the microscope using a calibrated eyepiece, and values were expressed in mm $\times 10^2$ as a specific migration distance by subtracting the control distance from the chemotactic distance. In each test a normal control blood sample was tested simultaneously with the patient's sample.

In vitro *survival of peripheral neutrophils* Different concentrations of leucocytes from rhG-CSF treated patients and a normal control, purified by density-gradient centrifugation of a leucocyte cell suspension over Lymphoprep separation medium (Nycomed) to deplete mononuclear cells, were plated with and without rhG-CSF ($3 \times 10^3$ U ml$^{-1}$) and live cell numbers were counted on a haemocytometer after staining with Trypan blue over a period of 6 days.

*Neutrophil alkaline phosphatase* The activity of this enzyme was determined daily during the phase I part of the study as described by Rutenberg (1965). A normal blood film and a blood film giving a strong reaction were used as controls.

### *Clonal assays for haemopoietic cells*

All methods were essentially as described before (Metcalf, 1977; Testa, 1985) with minor modifications.

*Cells* Bone marrow cells were obtained by aspiration from the posterior superior iliac crest before and after 5 days of continuous infusion of rhG-CSF (via central line) during the phase I part of our study. Cells were collected in Iscove's medium (Gibco) with 40 ml$^{-1}$ of preservative free heparin (sodium heparin Weddel Pharmaceuticals Ltd.) and red blood cells were separated by sedimentation in 0.1% methylcellulose over 30 min at room temperature.

*Clonogenic haemopoietic progenitor cell assay* Bone marrow cells were plated at $10^5$ cells ml$^{-1}$ in 0.9% methylcellulose, 20% conditioned medium from the 5637 carcinoma cell line (as a source of colony-stimulating factors, Myers *et al.*, 1984), 2 Units of partially purified urinary erythropoietin (Terry Fox Lab, Vancouver), with a final concentration of 1% bovine serum albumin (Sigma) and 10% foetal calf serum (FCS, Flow Lab) in Iscove's medium. Triplicate 1 ml aliquots were cultured in Multiwell™ tissue culture plates (Falcon) for 14 days under fully humidified conditions in an atmosphere of normal $O_2$ concentration at 37°C. Recognisable erythroid colonies were labelled as BFU-E (Burst Forming Unit-Erythroid) and myeloid colonies as GM-CFC (Granulocyte Macrophage-Colony Forming Cells) if >50 cells per aggregate. The ratio GM-CFC/BFU-E was calculated for each patient before and after the infusion of rhG-CSF and the results were analysed by the Wilcoxon matched-pairs signed-ranks test. All assays were performed with and without adherent bone marrow cells and in some experiments the *in vitro* response to rhG-CSF was tested by adding $3 \times 10^3$ U ml$^{-1}$ of rhG-CSF (AMGen) to the above medium mixture in the absence of conditioned medium. Adherent cell depletion was performed by incubating red blood cell depleted human bone marrow cells in Falcon tissue culture flasks at $10^6$ cells ml$^{-1}$ for 2 h at 37°C. Thereafter, the medium was collected and the cells counted and used for clonogenic assays as before.

*Tritiated thymidine suicide assay* $^3$H-TdR suicide, modified from Becker *et al.* (1965), was used to determine the percentage of clonogenic haemopoietic progenitor cells in the S-phase of the cell cycle. Paired 1 ml aliquots of $5 \times 10^6$ red cell depleted bone marrow cells were incubated for 30 min at 37°C in the presence of either 200 $\mu$Ci ml$^{-1}$ $^3$H-TdR, of specific activity 555 GBq mmol$^{-1}$ (Amersham), or an equal volume of medium. Incorporation of $^3$H-TdR was stopped by placing the cells on ice for 5 min, and washing them twice in Iscove's medium containing 10% FCS and 100 $\mu$g ml$^{-1}$ unlabelled thymidine. Thereafter, the cells were assayed for clonogenic cells by plating them at $1 \times 10^5$ cells in 1 ml of 0.3% agar that included Iscove's medium supplemented with a final concentration of 15% FCS and 20% conditioned medium from the 5637 bladder carcinoma cell line in 35 mm plastic Petri dishes (Falcon). Cultures were scored for colonies after 11 days of incubation under fully humidified conditions in an atmosphere of air plus 5% $CO_2$ at 37°C. The Standard Kill error was calculated as described by Lord & Schofield (1985) and results before and after the infusion of rhG-CSF were analysed by the Wilcoxon matched-pairs signed-ranks test.

### *Bone marrow trephines*

Bone marrow trephines were obtained under local anaesthetic from the posterior superior iliac crest before and after 5 days of continuous infusion of rhG-CSF (phase I) or when clinically indicated. They were fixed in 10% formol saline and decalcified overnight in 15% formic acid. Finally, they were processed to paraffin wax, cut and stained with hematoxylin and eosin. Trephines were examined at $\times 10$ magnification to determine marrow cellularity. An assessment of percentage of area occupied by haematopoietic tissue was made visually using coded slides. Replicate estimates generally gave results within 10% of each other.

Bone marrow aspirates were obtained at the same time as bone marrow trephines and smears were stained conventionally with May-Grumwald-Giemsa. The myeloid-erythroid ratios and differential counts were determined for each patient, and results analysed by the Wilcoxon matched-pairs signed-ranks test.

### Results

All 12 patients in our study responded to rhG-CSF with a specific increase in peripheral neutrophils (6 to 10-fold) up to a maximum of $100 \times 10^9$ l$^{-1}$ at 10 $\mu$g kg$^{-1}$ day$^{-1}$ of rhG-CSF. There were no appreciable changes in monocytes, eosinophils, platelets, lymphocytes or haemoglobin (Bronchud, *et al.*, 1987).

As shown in Figure 1, following infusion of rhG-CSF, there was a rapid but selective and transient fall in the number of peripheral neutrophils, followed by an increase 2 to 8 h later. We have shown previously (Bronchud *et al.*, 1987) that the increase in peripheral neurophils was maintained for as long as the infusion of rhG-CSF continued, but the counts fell back to normal levels within 24 to 48 h after stopping the growth factor infusion.

Table II shows that peripheral neutrophils after 3 days of rhG-CSF infusion were normal in tests of their mobility and phagocytic functions. The latter (as reflected by the chemiluminescence values) was usually increased (up to 2-fold) by rhG-CSF treatment and when it reached high values, neutrophil mobility appeared slightly reduced. Similar results were also obtained with peripheral neutrophils from rhG-CSF

**Table I** Patients study numbers, number of chemotherapy cycles received dose of rhG-CSF given, mean absolute neutrophil counts (ANC) at day 15, from day 1 of chemotherapy, *on* and *off* rhG-CSF infusion are shown. CT = chemotherapy, which consisted of i.v. adriamycin ($50\,mg\,m^{-2}$), i.v. etoposide $120\,mg\,m^{-2}$ ($\times 3$) and ifosfamide $5\,g\,m^{-2}$ (with mesna) given by i.v. infusion. Chemotherapy cycles were repeated every 3 weeks. Survival is measured from the date of histological diagnosis. PR = partial response; CR = complete response; NR = no response; ECR = equivocal complete response (minimal residual abnormalities on chest X-ray). Asterisks (*) denote patients still alive and on follow up

| Pt. | No. of CT cycles | Dose of rhG-CSF ($\mu g\,kg^{-1}\,day^{-1}$) | ANC at day 15 On G-CSF | ANC at day 15 Off G-CSF | Survival (months) | Response to CT |
|---|---|---|---|---|---|---|
| 1 | 4 | 1 | 12,000 | 211 | 5 | PR |
| 2 | 6 | 1 | 22,590 | 433 | 10 | CR |
| 3 | 6 | 5 | 72,224 | 295 | 10 | CR* |
| 4 | 6 | 5 | 24,323 | 110 | 9 | CR* |
| 5 | None | 10 | ND | | 5 | – |
| 5R | 4 | 10 | 36,000 | 64 | 12 | CR* |
| 6 | 3 | 10 | 18,900 | 616 | 3 | PR |
| 7 | None | 20 | ND | | 3 wks | – |
| 7R | 2 | 20 | 27,482 | 40 | 3 | PR (carcinoid) |
| 8 | None | 20 | ND | | 1 | – |
| 8R | 4 | 20 | 31,185 | 255 | 3 | NR |
| 9 | 4 | 40 | 34,000 | 200 | 7 | ECR* |

The number of infective complications requiring i.v. antibiotics were *ten* during the cycles of chemotherapy which did not include rhG-CSF, while only *one* infective episode occurred when patients were treated with rhG-CSF, and this was a bronchopneumonia in a non-neutropenic patient who had bronchial obstruction by tumour (8R).

**Figure 1** Composite figure showing time-response curves to rhG-CSF in patients 2 (●), 4 (▲), 8R (×) and 9 (■). Patients 8R and 9 (solid lines) had blood samples taken at zero time, 30 min, 1 h and 2 h after the start of the infusion of growth factor and illustrate the early fall in absolute neutrophil counts. Patients 2 and 4 (dashed lines) had blood samples taken at zero time, 2 h and later intervals and illustrate the increase in absolute neutrophil count. No changes were seen in other cell counts. A.N.C. = absolute neutrophil count $mm^{-3}$. Dose-response curves were shown in our previous paper (Bronchud *et al.*, 1987).

**Table II** Granulocyte function tests. Tests are shown for all 12 patients before (D0) and after 3 days (D3) of continuous infusion of the rhG-CSF. Peripheral neutrophils from patients 3 and 6 tested at the time of recovery from the nadir following chemotherapy showed chemiluminescence values of 2,542 and 2,731 respectively, with a migration distance of 71 and 40. The nominal range for chemiluminescence in our laboratory is wide (>750 mV) and for migration distance is 96–200 mm × $10^2$

| Pt. | Chemiluminescence (Units = mV) D0 | Chemiluminescence (Units = mV) D3 | Peak migration distance (mm × $10^2$) D0 | Peak migration distance (mm × $10^2$) D3 |
|---|---|---|---|---|
| 1 | 1,324 | 2,380 | 136 | 120 |
| 2 | 1,454 | 1,818 | 160 | 104 |
| 3 | 1,377 | 1,347 | 184 | 136 |
| 4 | 1,649 | 3,186 | 136 | 59 |
| 5 | 1,179 | 2,426 | 152 | 88 |
| 5R | 955 | 1,837 | 144 | 144 |
| 6 | 1,829 | 2,552 | 168 | 104 |
| 7 | 1,697 | 3,481 | 184 | 96 |
| 8 | 1,296 | 2,230 | 120 | 160 |
| 8R | 1,324 | 3,439 | 152 | 96 |
| 9 | 2,306 | ND | 152 | ND |

treated patients (patients 3 and 6) at the time of recovery from the nadir following chemotherapy (day 15). Their protective effect *in vivo* was suggested by the observation that the number of infective complications requiring i.v. antibiotics were *ten* during the cycles of chemotherapy which did not include rhG-CSF, with positive blood cultures in three patients, while only *one* infective episode occurred when patients were treated with rhG-CSF, and this was a bronchopneumonia in a non-neutropenic patient who had not responded to chemotherapy (Bronchud, *et al.*, 1987; and Table I).

We decided to investigate whether rhG-CSF can confer an increased survival to peripheral neutrophils (Figure 2). In this experiment peripheral blood leucocytes obtained from patients during rhG-CSF treatment exhibited similar survival kinetics, with or without added rhG-CSF, to those obtained from normal volunteers when cultured *in vitro*. However, we also found that when peripheral blood leucocytes were cultured from patients who had received rhG-CSF, the numbers of polymorphs recovered after 24 h was higher than the original value (Figure 2). This presumably reflects *contamination* of the peripheral blood leucocytes with a significant number of proliferatively active myeloid precursor cells as a result of the *in vivo* treatment with rhG-CSF. In

**Figure 2** *In vitro* survival of peripheral leucocytes. Peripheral leucocytes from a patient treated with rhG-CSF (solid lines) and from a normal control (dashed lines) were incubated *in vitro* at different cell concentrations in the presence (full symbols) or absence (open symbols) of added rhG-CSF ($3 \times 10^3$ U ml$^{-1}$). Cells were plated at the following concentrations: □: $4 \times 10^6$ ml$^{-1}$, ○: $2 \times 10^6$ ml$^{-1}$, Δ: $10^6$ ml$^{-1}$. The patient's differential count at zero time showed 4% myelocytes.

fact, most patients receiving 10–40 μg kg$^{-1}$ day$^{-1}$ rhG-CSF had a median of 4% of peripheral metamyelocytes (range 1–15%) after ~48 h and 2% myelocytes (range 1–4%) after 4 days of growth factor infusion. More importantly, however, no blast cells were ever seen. This *contamination* of peripheral blood leucocytes by immature forms might also help to explain the reduction in neutrophil mobility found in patients 3 and 6 on recovery from the nadir induced by chemotherapy, since both patients had significant numbers of immature forms at the time of sampling (10% and 14% respectively).

The neutrophil alkaline phosphatase activity also increased in all patients after ~48 h of rhG-CSF infusion, up to a maximum of 389 at 10 μg kg$^{-1}$ day$^{-1}$ (normal range is 15–100).

Another measure of dynamic changes in response to rhG-CSF was shown by the hypercellularity seen in bone marrow trephines of all patients following growth factor treatment during the phase I part of the study (Figure 3). Coded samples taken before and after the infusion of growth factor could be easily distinguished, and the median absolute increase in cellularity was 20% (from 30% pre-therapy to 50% post). A bone marrow trephine was also obtained from patient 4 when rhG-CSF was given after the sixth and last cycle of chemotherapy and showed a comparable degree of hypercellularity at the time of recovery from the chemotherapy induced neutrophil nadir. The proportion of cycling haemopoietic progenitor cells (Table III) was only slightly increased by rhG-CSF treatment (median of 8.5%, $P = 0.028$).

The GM-CFC/BFU-E ratio was not statistically different ($P = 0.25$) after 5 days of rhG-CSF infusion (Table III). However, the myeloid-erythroid ratio in bone marrow aspirates was significantly raised ($P = 0.028$), up to 5-fold pre-treatment values, by the growth factor infusion (Table IV).

We found no evidence of a stimulatory effect of rhG-CSF on small cell lung cancer. Thus a repeat bone marrow

**Figure 3** Staining of bone marrow trephines from patient 4 before (a) and after (b) 5 days of rhG-CSF infusion at 5 μg kg$^{-1}$ day$^{-1}$. (H&E, ×6.3).

**Table III** Bone marrow *in vitro* assays. Haemopoietic progenitor cells in S-phase (%) (in 0.3% agar with 15% FCS and 20% conditioned medium) and GM-CFC/BFU-E ratios (in 0.9% methylcellulose with 10% FCS, 20% conditioned medium and 2 U erythropoietin) are shown before (D0) and after (D5) 5 days of continuous infusion of rhG-CSF. The values shown as % of haemopoietic progenitor cells in S-phase are the calculated mean; the standard kill error was < ±5% for all tests. The range of % of haemopoietic progenitor cells in S-phase for 3 normal controls was 35–49%. Asterisks (*) denote bone marrows histologically involved by small cell lung cancer

| | *% in S-phase* | | *GM-CFC/BFU-E ratio* | |
|---|---|---|---|---|
| *Pt.* | *D0* | *D5* | *D0* | *D5* |
| 1 | 63 | 73 | 0.71 | 4.0 |
| 2 | 43 | 49 | 1.6 | 2.1 |
| 3 | 50 | 68 | 0.88 | 1.9 |
| 4* | 40 | 47 | 2.2 | 2.8 |
| 5 | 85 | ND | 3.3 | ND |
| 5R* | 64 | 69 | 1.26 | 2.2 |
| 6 | 49 | ND | 1.8 | ND |
| 7* | 59 | 73 | 4.8 | 2.6 |
| 8* | 43 | ND | 3.2 | ND |

**Table IV** Myeloid-erythroid ratios in bone marrow aspirates from patients 1–6 before and after 5 days of rhG-CSF infusion

| | *rhG-CSF treatment* | |
|---|---|---|
| *Pt.* | *Pre-* | *Post-* |
| 1 | 5.5/1 | 6.8/1 |
| 2 | 3.2/1 | 5.8/1 |
| 3 | 4.2/1 | 4.4/1 |
| 4 | 5.5/1 | 26.3/1 |
| 5 | 3.6/1 | 7.2/1 |
| 6 | 3.1/1 | 5.1/1 |

trephine in one of our patients (performed on the same site), with pre-treatment extensive replacement by small cell carcinoma cells, revealed no malignant cells following chemotherapy and rhG-CSF treatment. Although the numbers are obviously small, a complete remission rate of 50% (Table I) compares favourably with the best published series for advanced small cell lung cancer (Spiro, 1985). The median survival of the 8 evaluable patients with histologically proven advanced small cell lung cancer is now 8 months and 4 patients remain alive and on follow up (Table I). Small cell carcinoma cells were grown *in vitro* from the bone marrow of another patient, but did not respond to the presence of rhG-CSF in the medium and they died within 10 days.

One patient died during the study while receiving rhG-CSF (8R) and microscopic evidence of some extramedullary haemopoiesis in the spleen was found at post-mortem.

### Discussion

Our results suggest that rhG-CSF *in vivo* has at least two main effects: (a) an early fall in peripheral neutrophils within the first hour, followed by a rapid *influx* of mature neutrophils into the circulatory pool; (b) stimulation of proliferation and differentiation of neutrophil precursors in the bone marrow.

The early *influx* occurs between 2 and 8 hours after the start of the continuous infusion of rhG-CSF (Figure 1) and is specific for neutrophils. Initially, only mature neutrophils are released into the circulation, but at high doses of rhG-CSF some metamyelocytes and myelocytes are also seen in the peripheral blood after 2 or 3 days. This response is quite different from the biphasic leucocyte changes which are seen following the parenteral administration of epinephrine (Samuels, 1951) which causes a prompt mobilisation of all white cell elements (maximal within 20 minutes) thought to be secondary to demargination of leucocytes from post-capillary venules. In fact, during the first hour of the rhG-CSF infusion we found a transient decrease in peripheral neutrophils with no significant change in other circulating cell numbers. The reason for this initial decrease is unclear and requires further investigation, but it might be related to migration of circulating neutrophils into tissues or to increased adherence of neutrophils to endothelium. Similar findings have been recently reported by Morstyn *et al.* (1988) following rhG-CSF given by short intravenous infusion. The subsequent rise in peripheral neutrophils produced by rhG-CSF is probably due to an increase in the *influx* of cells from the bone marrow (both mature and *de novo* granulocytes). This is similar to one of the mechanisms proposed for cortisone-induced neutrophil leucocytosis, but the latter is also known to result from a decreased efflux of cells from the blood (Bishop *et al.*, 1968), and it is not associated with a net increase in the rate of bone marrow neutrophil production (Vincent, 1977).

It is possible that part of the explanation for the increased neutrophil count is due to prolonged survival of myeloid cells in the presence of rhG-CSF. Our analysis *in vitro* suggests that the growth factor does not significantly prolong survival of either control cells or cells obtained from patients treated with growth factor. However, a marginal increase in neutrophil survival *in vitro*, between 24 and 40 hours, has been reported by Begley *et al.* (1986) when neutrophils were plated at much lower densities in the presence of purified murine G-CSF. We did not look for such effect. The finding that peripheral neutrophil counts in patients return to normal levels within 24–48 hours after stopping the growth factor infusion also agrees with the best estimates of the normal half-life of circulating neutrophils *in vivo* (about 8 hours, Vincent, 1977), as one would expect a minimum of 4 half-lives to elapse before blood counts return to normal by a process of gradual cell loss.

The increased cellularity seen after 1 day following culture of cells from patients treated with rhG-CSF probably reflects the presence of proliferating precursor cells in peripheral blood at the time of sampling, and does not appear to be influenced by the addition of rhG-CSF to the *in vitro* cultures. Thus, while the initial increase in peripheral neutrophils *in vivo* almost certainly reflects a release of myeloid cells from the bone marrow, the *sustained* increase presumably reflects increased proliferative activity in the bone marrow, rather than more prolonged survival of circulating cells.

Indeed, this stimulation of haemopoiesis *in vivo* in the bone marrow was directly demonstrated by the 20% increase in bone marrow cellularity seen in bone marrow trephines after the infusion of growth factor and by a significant increase in the myeloid-erythroid ratio in bone marrow aspirates. Bone marrow cellularity at the time of recovery from the chemotherapy induced neutrophil nadir (day 15) when rhG-CSF was given after the sixth and last chemotherapy cycle was similar to that observed after the infusion of growth factor during the phase I part of the study. Most of the expansion in cell numbers probably occurs after the GM-CFC stage, as the increase in the proportion of cycling haemopoietic progenitor cells was only small (median of 8.5%). However, we cannot exclude a fractional stimulatory effect of rhG-CSF on GM-CFC cells in patients, as the GM-CFC/BFU-E ratio was often slightly increased after growth factor treatment, although this increase did not reach statistical significance. Of course, the presence of other factors, such as interleukin-1(IL-1), in patients with advanced lung cancer is likely, and the known interactions between IL-1 and rhG-CSF (Moore & Warren, 1987) preclude any simplistic interpretation in the *in vivo* effects of rhG-CSF.

The median survival of the eight evaluable patients with histologically proven advanced small cell lung cancer in this study is now 8 months and four patients remain alive and on follow up. This survival is consistent with that of currently employed chemotherapy regimes (Aisner, 1987).

Our results in patients suggest that, when given as a continuous infusion at 1–40 $\mu g\,kg^{-1}\,day^{-1}$, rhG-CSF results in a specific increase in peripheral neutrophils and neutrophil precursors.

We thank the Cancer Research Campaign and the Leukaemia Research Fund for their support. We would also like to thank M. Jones for the statistical analysis of data and AMGen Inc., Thousand Oaks, California for supplying rhG-CSF.

### References

AISNER, J. (1987). Identification of new drugs in small cell lung cancer. *Cancer Treat. Rep.*, **71**, 1131.

BECKER, A.J., McCULLOCH, E.A., SIMINOVITCH, L.L. & TILL, J.E. (1965). The effect of differing demands for blood cell production on DNA synthesis by hemopoietic colony forming cells of mice. *Blood*, **26**, 296.

BEGLEY, C.G., LOPEZ, A.F., NICOLA, N.A. & 4 others (1986). Purified colony-stimulating factors enhance the survival of human neutrophils and eosinophils *in vitro*: A rapid and sensitive microassay for colony-stimulating factors. *Blood*, **68**, 162.

BISHOP, C.R., ATHENS, J.W., BOGGS, D.R., WARNER, H.R., CARTWRIGHT, G.E. & WINTROBE, M.M. (1968). A non-steady kinetic evaluation of the mechanism of cortisone-induced granulocytosis. *J. Clin. Invest.*, **47**: 249.

BRONCHUD, M.H., SCARFFE, J.H., THATCHER, N. & 5 others (1987). Phase I/II study of recombinant human granulocyte colony-stimulating factor in patients receiving intensive chemotherapy for small cell lung cancer. *Br. J. Cancer*, **56**, 809.

DONAHUE, R.E., WANG, E.A., STONE, D.K. & 5 others (1986). Stimulation of haematopoiesis in primates by continuous infusion of recombinant human GM-CSF. *Nature*, **321**, 872.

EASMON, C.S.F., COLE, P.J., WILLIAMS, A.J. & HASTINGS, M. (1980). The measurement of opsonic and phagocytic function by luminol-dependent chemiluminescence. *Immunology*, **41**, 67.

FLEISCHMANN, J., GOLDE, D.W, WEISBART, R.H. & GASSON, J.C. (1986). Granulocyte-macrophage colony-stimulating factor enhances phagocytosis of bacteria by human neutrophils. *Blood*, **68**, 708.

GROOPMAN, J.E., MITSUYASU, R.T., DE LEO, M.J., OETTE, D.H. & GOLDE, D.W. (1987). Effect of recombinant human granulocyte colony-stimulating factor on myelopoiesis in the acquired immunodeficiency syndrome. *New Engl. J. Med.*, **317**, 593.

LORD, B.I. & SCHOFIELD, R. (1985). Haemopoietic spleen colony-forming units. In *Cell Clones: Manual of Mammalian Cell Techniques*, Potten, C.S. & Henry, J.H. (eds) p. 13. Churchill Livingstone: New York.

METCALF, D. (1977). *Hemopoietic Colonies*. Springer-Verlag: New York.

METCALF, D. (1986). The molecular biology and functions of the granulocyte-macrophage colony-stimulating factors. *Blood*, **67**, 257.

MOORE, M.A.S. & WARREN, D.J. (1987). Synergy of interleukin 1 and granulocyte colony-stimulating factor: *In vivo* stimulation of stem-cell recovery and hematopoietic regeneration following 5-fluorouracil treatment of mice. *Proc. Natl Acad. Sci.*, **84**, 7134.

MORSTYN, G., CAMPBELL L., SOUZA, L.M. & 6 others (1988). Effect of granulocyte colony stimulating factor on neutropenia induced by cytotoxic chemotherapy. *Lancet*, **i**, 667.

MYERS, C.D., KATZ, F.E., JOSHI, G. & MILLAR, J.L. (1984). A cell line secreting stimulating factors for CFU-GEMM culture. *Blood*, **64**, 152.

NELSON, R.D., QUIE, P.G. & SIMMONS, R.L. (1975). Chemotaxis under agarose: A new and simple method for measuring chemotaxis and spontaneous migration of polymorphonuclear leukocytes and monocytes. *J. Immunol.*, **15**, 1650.

NICOLA, N.A. (1987). Granulocyte colony-stimulating factor and differentiation-induction in myeloid leukemic cells. *Int. J. Cell Clon.*, **5**, 1.

RUTENBURG, A.M., ROSALES, C.L. & BENNETT, J.M. (1965). An improved histochemical method for the demonstration of leucocyte alkaline phosphatase activity: Clinical application. *J. Lab. and Clin. Med.*, **65**, 698.

SAMUELS, A.J. (1951). Primary and secondary leucocyte changes following intramuscular injection of epinephrine hydrochloride. *J. Clin. Invest.* **30**, 941.

SIEFF, C.A. (1987). Hematopoietic growth factors. *J. Clin. Invest.*, **79**, 1549.

SOUZA, L.M., BOONE, T.C., GABRILOVE, J. & 12 others (1986). Recombinant human granulocyte colony-stimulating factor: Effects on normal and leukaemic myeloid cells. *Science*, **232**, 61.

SPIRO, S.G. (1985). Chemotherapy of small lung cancer. In *Clinics in Oncology*, **4**, Spiro, S.G. (ed) p. 105. W.B. Saunders Co.: London.

TESTA, N.G. (1985). Clonal assays for haemopoietic and lymphoid cells *in vitro*. In *Cell Clones: Manaual of Mammalian Cell Techniques*, Potten, C.S. and Henry, J.H. (eds) p. 27. Churchill Livingstone: New York.

VADHAN-RAJ, S., KEATING, M., LE MAISTRE, A. & 6 others (1987). Effects of recombinant human granulocyte-macrophage colony-stimulating factor in patients with myelodysplastic syndromes. *New Engl. J. Med.*, **317**, 1545.

VINCENT, P.C. (1977). Granulocyte kinetics in health and disease. In *Clinics in Haematology*, **6**, Lewis, S.M. (ed) p. 695. W.B. Saunders Co. Ltd.: London.

WELTE, K., BONILLA, M.A., GILLIO, A.P. & 6 others (1987). Recombinant human granulocyte colony-stimulating factor. *J. Exp. Med.*, **165**, 941.